# Cytotoxicity and Apoptosis Induction of* Ardisia crispa* and Its Solvent Partitions against* Mus musculus* Mammary Carcinoma Cell Line (4T1)

**DOI:** 10.1155/2017/9368079

**Published:** 2017-03-16

**Authors:** Muhammad Luqman Nordin, Arifah Abdul Kadir, Zainul Amiruddin Zakaria, Fauziah Othman, Rasedee Abdullah, Muhammad Nazrul Hakim Abdullah

**Affiliations:** ^1^Department of Veterinary Preclinical Sciences, Faculty of Veterinary Medicine, Universiti Putra Malaysia (UPM), 43400 Serdang, Selangor, Malaysia; ^2^Department of Biomedical Sciences, Faculty of Medicine and Health Science, Universiti Putra Malaysia (UPM), 43400 Serdang, Selangor, Malaysia; ^3^Department of Human Anatomy, Faculty of Medicine and Health Sciences, Universiti Putra Malaysia (UPM), 43400 Serdang, Selangor, Malaysia; ^4^Prof. Fauziah Biohealth Sdn. Bhd, Malaysia; ^5^Department of Veterinary Pathology and Microbiology, Faculty of Veterinary Medicine, Universiti Putra Malaysia (UPM), 43400 Serdang, Selangor, Malaysia

## Abstract

This study was conducted to investigate the cytotoxicity and apoptosis effect of* A. crispa* extract and its solvent partition (ethyl acetate and aqueous extract) against* Mus musculus* mammary carcinoma cell line (4T1). The normal mouse fibroblast cell line (NIH3T3) was used as comparison for selective cytotoxicity properties. The cytotoxicity evaluation was assessed using MTT assay. AO/PI dual fluorescent staining assay and Annexin V-FITC were used for apoptosis analysis. Results showed that 80% methanol extract from leaves showed most promising antimammary cancer agent with IC50 value of 42.26 ± 1.82 *μ*g/mL and selective index (SI) value of 10.22. Ethyl acetate was cytotoxic for both cancer and normal cell while aqueous extract exhibited poor cytotoxic effect. 4T1 cells labelled with AO/PI and Annexin V-FITC and treated with 80% methanol extract demonstrated that the extract induces apoptosis to 4T1 mammary cancer cells. In conclusion, 80% methanol extract of* A. crispa* was selectively cytotoxic towards 4T1 cells but less cytotoxic towards NIH3T3 cells and induced the cancerous cells into apoptotic stage as early as 6 hours.

## 1. Introduction

*Ardisia crispa* (Thunb.) A. DC., a family member of Myrsinaceae, is a plant that is widely distributed over Asian countries including Malaysia, Japan, India, and Indonesia. This plant is known by variety of names such as Coral Ardisia, Coral Bush, Christmas Berry, and Hen's eyes. Local Malaysian people often call this plant “pokok Mata Ayam.” Traditionally, the roots of the plant were boiled and have been used for herbal preparation for treatment of numerous human ailments. The roots of this plant species were claimed by traditional practitioners to possess numerous therapeutic benefits such as ability to treat fever, rheumatism, cough, diarrhoea, menstrual pain, pain, insect bites, and swelling and to improve general blood circulation. Scientific studies conducted mostly focus on the root of the* A. crispa* and there is still little information about the effect from other parts of the plant such as from its leaves. Previous scientific studies have demonstrated numerous biological activities of* A. crispa* particularly in its root part including antipyretic [1], anti-inflammatory and antihyperalgesic [2, 3], antiulcer [4], and anticancer (skin and liver) activities [5–7]. Mammary cancer is the most leading cancer among females globally [8]. Mammary cancer can affect animal and human and it is cancer that arises from mammary gland. In human, due to anatomical location of mammary gland in breast, it is called breast cancer. However, the incidence of mammary cancer in males is extremely low (less than 1%). Indeed, many studies have been conducted worldwide in order to discover the new potential source of anticancer and medicinal plants seem to provide a source of anticancer agents [3, 9].

*Mus musculus* mammary carcinoma (4T1) cell is a type of animal model of mammary carcinoma and can be induced in BALB/c mice. This type of cancer cell has gained attention from many researchers because it closely mimics stage IV human breast cancer and an alternative study about human breast cancer using animal model of cancer cell. However, to the best of our knowledge, no one has conducted any scientific evaluations of* A. crispa* on malignant mammary cancer and normal cell with the mode of cancer death. Previous studies particularly focused on root part of the plant and none reported on the leaves. Now, the aim of this study is to evaluate the anticancer potentials from the leaves of* A. crispa. *

## 2. Materials and Methods

### 2.1. Collection and Identification

*A. crispa* leaves were collected from Biodiversity Unit, Universiti Putra Malaysia, and certified by botanist of the Institute Bioscience (IBS), Universiti Putra Malaysia (UPM), Serdang, Selangor, Malaysia, by comparing with deposited specimens (SK 2834/15) from Herbarium of Natural Products, IBS, UPM. The leaves were cleaned and then dried in oven at 37°C for 7 days.

### 2.2. Preparation of Extracts

Leaves from* A. crispa* were cleaned with tab water and dried in oven for a week. The leaves then pulverized using commercial blender (model HGB2WTS3). Pulverized leaves (200 g) were soaked with 80% methanol (hydromethanol extraction) for 72 hours at room temperature. The solvent-containing extracts were evaporated under reduced pressure by using a vacuum rotary evaporator (Heidolph Germany) and controlled heating bath at 30°C. The yield that was obtained was further dissolved and extracted with ethyl acetate and aqueous extract in Soxhlet extractor. The yield that was obtained was then stored at −20°C until used for analysis.

### 2.3. Cell Line and Culture Medium

*Mus musculus* mammary carcinoma cancer cell line (4T1) was gleaned from American Type Culture Collection (ATCC), USA. The cells were cultured in Roswell Park Memorial Institute (RPMI-1640) medium without phenol red. All the media were supplemented with 10% fetal bovine serum (FBS), 1% antibiotic-antimycotic (Gibco®, Thermo Fisher Scientific) as a complete growth medium (CGM). The cells were thawed gradually from liquid nitrogen to −80°C freezer and then 37°C water bath prior to culture with 5% carbon dioxide (CO_2_) in incubator at 37°C. The cells were checked daily for viability, proliferation, and confluency.

### 2.4. In Vitro Cytotoxicity Assay

The confluent 4T1 cells were detached and harvested with 0.25% trypsin (1x). Briefly, 100 *μ*L of media containing 1 × 10^4^ cells was seeded in 96-well plates. The plate was incubated overnight to allow cell attachment to the bottom of the well plates. After overnight incubation, old media were removed. The extracts were dissolved in dimethyl sulfoxide (DMSO) with the highest concentration of DMSO which is 0.2%. The cells then were treated with extracts at concentration of 1000, 500, 250, 125, 62.5, 31.25, and 15.63 *μ*g/mL. Each concentration of treated cells, untreated cells (0 *μ*g/mL), and blank was performed in triplicate in one plate and the experiment was repeated for three times for validity. The plates were further incubated for 72 hours. After 72 hours of treatment period, the old media were removed and replaced with 100 *μ*L of fresh medium. 20 *μ*L of filtered MTT solution (5 mg/mL PBS) was added to each well. The plates were wrapped with aluminium foil and incubated again for 3 hours at 37°C with 5% CO_2_. Carefully, 110 *μ*L medium was aspirated from each well and, then, 100 *μ*L of DMSO was added to each well and mixed thoroughly with pipette. The plates were reincubated for another 10 minutes. The optical density (OD) was measured using spectrophotometer (Infinite M200 PRO) at 570 nm after 10 minutes of incubation.

The percentage of cell viability (%) was calculated using the formula:

(1) Cell viability %=mean OD of treated cell−mean OD of blankmean OD of untreated cell−mean OD of blank×100

The cytotoxic effects against 4T1 cells after 72 hours were recorded as IC_50_ compared with untreated cells [10, 11]. The percentages of cell viability values against concentration of respective extracts were plotted in order to determine the IC_50_ values of each extract. The IC_50_ value refers to the concentration of the extract able to inhibit 50% of the cells population. The comparison between treated cells and untreated cell was evaluated by using one-way ANOVA followed by Dunnett's multiple comparison tests. Significant difference was considered accepted at *P* < 0.05.

### 2.5. Selectivity Index of* A. crispa* and Its Partitions

The selectivity indexes (SI) of HEAC, EAEAC, and AQEAC were estimated according to the method described in [12] with slight modification which were as follows:

(2) $$\begin{array}{c} SI=\frac{{CC}_{50}}{{IC}_{50}}, \end{array}$$

where SI is selectivity index. CC_50_ is minimum cytotoxic concentration that allows at least 50% cells to survive. IC_50_ is minimum inhibitory concentration that kills at least 50% of cells.

Normal mouse fibroblast cell line (NIH3T3) was used as comparison for extract with 4T1 mammary cancer cells. The NIH3T3 cell line was gleaned from ATCC and was grown in RPMI 1640 medium with L-glutamine, supplemented with 10% fetal bovine serum (FBS) (Gibco, 1% antibiotic-antimycotic) (10,000 units/mL of penicillin, 10,000 *μ*g/mL of streptomycin, and 25 *μ*g/mL amphotericin B) as a complete growth medium (CGM).

### 2.6. Phytochemical Screenings

The test was conducted to detect the presence of phytochemical constituents of the extracts including phenolics, flavonoids, alkaloids, saponins, tannins, and terpenoids which are well known to have multiple biological properties such as antioxidant, antimicrobial, and anticancer properties [13–15].

#### 2.6.1. Detection of Phenolics

Five milliliters of sterile distilled water was added to 0.5 mL of HEAC, EAEAC, and AQEAC in test tubes. Five drops of 1% ferric chloride (FeCl_3_) solution were added to the test tubes and allowed to mix. The formation of bluish black solution indicates presence of phenols [16].

#### 2.6.2. Detection of Flavonoids

The flavonoids detection was based on alkaline reagent test described in [17] with slight modification. Three milliliters of HEAC, EAEAC, and AQEAC was mixed with 1 mL of 10% sodium hydroxide (NaOH) solution in test tubes. Development of intense yellow, which becomes colourless on addition of dilute acid, indicates the presence of flavonoids.

#### 2.6.3. Detection of Saponins

The saponins detection was based on froth test described in [16]. Half milliliters extract was mixed with 2 mL of distilled water in test tube and then mixed thoroughly. The presence of saponins was detected by consistence of the froth formation for 10 minutes.

#### 2.6.4. Detection of Tannins

The tannins detection was based on method described in [18]. 0.5 mL of HEAC, EAEAC, and AQEAC were dissolved in 20 mL distilled water in a test tube. The mixed solutions were filtered with Whatman Number 1 filter paper. The 2 mL of filtrated solutions was treated with 1% ferric chloride (FeCl_3_) solution. Blackish-blue or blackish green indicates the presence of tannins.

#### 2.6.5. Detection of Terpenoids

Terpenoids detection was based on method described in [19]. 0.5 mL of HEAC, EAEAC, and AQEAC was mixed with 3 mL of chloroform. Then 3 mL of concentrated sulfuric acid (H_2_SO_4_) was carefully added drop by drop to each extract to form layers. Positive availability is when reddish brown colouration layer is formed.

### 2.7. Acridine Orange/Propidium Iodide (AO/PI) Dual Fluorescent Staining Assay

The presence of apoptosis event was observed using acridine orange/propidium iodide (AO/PI) dual fluorescent staining assay. 2 × 10^5^ 4T1 mammary cancer cells were seeded into two 25 cm^2^ sterile flasks and 4 mL of complete media was added. One of the flasks was a control (untreated cells). The flasks were incubated overnight in incubator at 37°C with 5% CO_2_ supplementation. The next day, the old media in one flask were aspirated out, followed with washing with 5 mL of phosphate buffer saline (PBS) and replaced with new media with treatment. In another flask, the same procedure was repeated but 4 mL of new media was added without treatment. Both flasks were incubated for 72 hours at 37°C with 5% CO_2_ supplementation. The concentration of treatment was based on the IC_50_ of 80% methanol extract. After 72 hours of treatment time, the old media were aspirated into 15 mL centrifuge tubes. The flasks were washed with cold PBS slowly and then were aspirated into the same centrifuge tubes. The attached cells in flasks also were detached with 0.25% EDTA trypsin and the detached cells were aspirated into the same 15 mL centrifuge tube containing the old media, PBS, and trypsinized cells. The collected cells in 15 mL centrifuge tube were centrifuged at 1200 rpm for 5 minutes. The supernatants were aspirated out. Ten microliters (10 mg/mL) of acridine orange (AO) and 10 *μ*L (1 mg/mL) of propidium iodide (PI) were added to centrifuge tube and resuspended. The mixtures were incubated for 10 minutes in dark room at room temperature. Finally, after 10 minutes of incubation time, 10 *μ*L of mixture solution was aspirated and placed onto microscope slide and covered with coverslip. The slides were viewed under inverted research fluorescence microscope (Nikon ECLIPSE T*i*-S, Shinagawa-ku, Tokyo, Japan) at 20x magnification.

### 2.8. Quantitative Apoptosis Analysis Using Flow Cytometer

5 × 10^5^ 4T1 mammary cancer cells were seeded into 25 cm^2^ sterile flasks and 4 mL of complete media was added to the flasks to allow cells attachment overnight. The next day, the old media were removed and the cells were treated with HEAC according to its IC_50_ concentration and further incubated for 6 and 24 hours. The detection of apoptotic cells was performed based on protocol described by ApoDetect™ Annexin V-FITC Kit (Invitrogen), catalogue number 33-1200. After 6 and 24 hours of incubation times, the old media were aspirated by serological pipette onto 15 mL centrifuge tube. The flasks then were washed with cold PBS and the liquids were aspirated again onto the same centrifuge tube. One milliliter of 0.25% EDTA trypsin was added to the flasks to detach the remaining cells. The trypsinized cells were aspirated and collected to the same centrifuge tubes. The centrifuge tubes then were spun down at 3000 rpm for 1 minute. The supernatants were gently removed. Briefly, 100 *μ*L binding buffer was added and mixed gently to the cells by tapping with hand. Ten microliters of Annexin V-FITC and 10 *μ*L propidium iodide (20 *μ*g/mL) were added to cells suspension and resuspended gently. The mixtures were incubated 10 minutes at room temperature. Then, the cells were washed with 400 *μ*L binding buffer, sequentially centrifuged at 3000 rpm for 1 minute. The supernatants were removed and 200 *μ*L binding buffer was added again. The samples were filtered through 50 *μ*L nylon mesh before being transferred into 12 × 75 mm tubes. The apoptosis analyses of treatment and control samples were run using flow cytometry 10,000 cells per event.

### 2.9. Statistical Analysis

All the percentages of cell viability were expressed as mean (*n* = 3) per plate ± SD (standard deviation) and differences among treated and untreated cells were analysed using one-way ANOVA followed by Dunnett's multiple comparison test. The test was considered statistically significant when *P* < 0.05 as compared to untreated cell (control) and GraphPad Prism Software 5.0 was used to analyse all statistical tests.

## 3. Results

**Figure 1:**
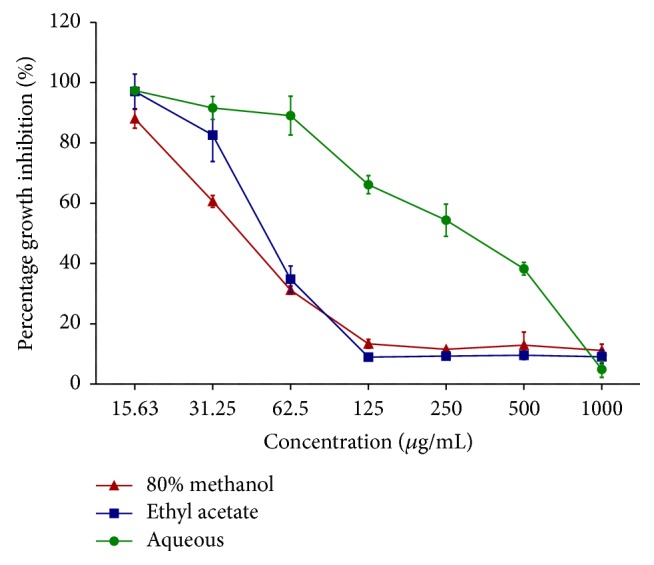
Growth inhibition of* A. crispa* leaves extract on 4T1 mammary carcinoma cell line after 72 hours of treatment. Every point denotes mean (*n* = 3) of triplicate sample. Error bars indicate standard deviation.

**Figure 2:**
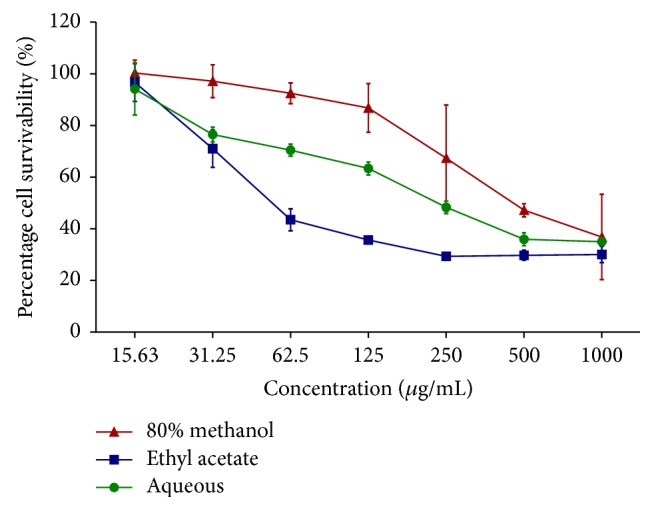
Cell survivability of* A. crispa* leaves extracts on normal mouse fibroblast cell line (NIH3T3) after 72 hours of treatment. Every point denotes mean (*n* = 3) of triplicate sample. Error bars indicate standard deviation.

Table 1 represents the percentage (%) of cell viability of leaves extract of* A. crispa* on 4T1 mammary cancer cell lines while Table 2 represents the percentage (%) of cell survivability of leaves extract of* A. crispa* on normal mouse fibroblast cell line (NIH3T3) after 72 hours of treatment, respectively. The cell viability of 4T1 against treatment with* A. crispa* and its solvent partitions were assessed using MTT assay. The inhibitory concentration (IC_50_) and survivability (CC_50_) of the cell versus various extract treatment were constructed through linear regression principle (Figures 1 and 2). The IC_50_ and CC_50_ values of 80% methanol extract, ethyl acetate extract, and aqueous extract were shown in Table 3. The results demonstrated that 80% extract shows the highest inhibitory level on cancer cell and survivability rate on normal cell, making it able to be considered as a potential antimammary cancer agent.

The phytochemical screening results of 80% methanol extract and its partitions (ethyl acetate and aqueous extract) are displayed in Table 4. Phytochemical constituents, namely, phenolics, flavonoids, saponins, tannins, and terpenoids, were determined with various standard screening tests. Overall, 80% methanol extract, ethyl acetate extract, and aqueous extract contain all the phytochemicals listed in Table 4 except for saponins in aqueous extract.

### 3.1. Apoptosis Detection with Dual Fluorescent Staining

**Figure 3:**
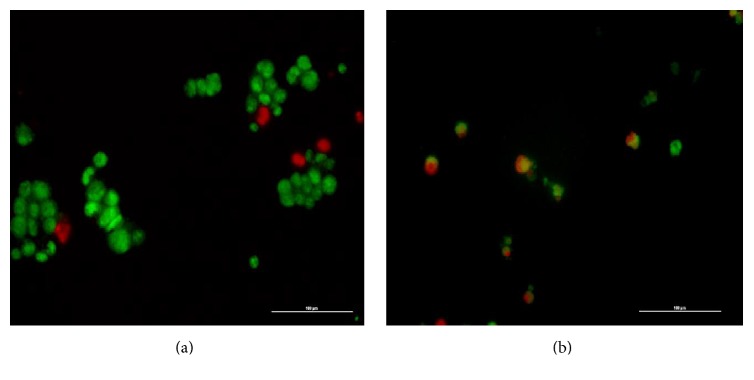
4T1 cells stained with dual staining AO/PI following treatment with 80% methanol extract for 72 hours. The cells were viewed under inverted research fluorescence microscope (Nikon ECLIPSE T*i*-S, Shinagawa-ku, Tokyo, Japan) at 20x magnification. (a) Control (untreated 4T1 cells) being majorly viable and uniform in shape. Green fluorescent colour indicates cells are viable. (b) 4T1 cells treated with 42.26 *μ*g/mL of 80% methanol extract. Red colour indicates cells that had undergone necrosis while orange colour indicates cells that had undergone apoptosis. Number of viable cells was reduced and cells were showing shrinkage. Scale bars represent 100 *μ*m.

Figure 3 is the representative picture of the treated and untreated 4T1 mammary cancer cells after 72 hours of treatment with IC_50_ value from 80% methanol extract, stained with dual staining acridine orange/propidium iodide (AO/PI). Based on MTT assay, it showed that 80% ethanol extract is the best extract and the IC_50_ against 4T1 mammary cancer cells was the lowest where IC_50_ value is 42.26 *μ*g/mL. Therefore, AO/PI dual fluorescent staining assay was performed on 4T1 mammary cancer cell line. Majority of untreated cells emitted green fluorescence colour. The treated cells emitted red fluorescence colour indicating that the cell death is due to necrosis event, whereby the PI dye remains bound to the nucleated dead cells. The green fluorescence colour is indicating that the cell is alive and the AO dye permeable to it, whereas orange colour is indicating apoptotic cells due to apoptosis event. Besides the colour emission from the staining, the morphology, number, and size of the cells were also determined before and after treatment. For untreated cells, the cells were uniform in size and morphology. For treated cells, the size was not uniform. The number of cells also reduced and the morphological appearance of the cells was showing shrinkage.

### 3.2. Quantitative Apoptosis Analysis Using Flow Cytometer

**Figure 4:**
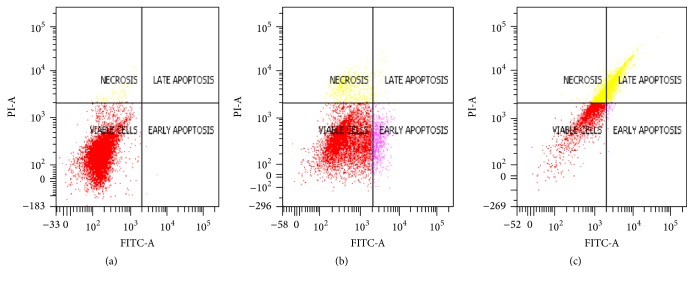
Apoptosis event occurs following treatment with 80% methanol extract at their respective IC_50_ after 6 and 24 hours. (a) Control (untreated cells) with no apoptosis event occurrence. (b) and (c) are treated cells after 6 and 24 hours, respectively. The apoptosis event occurs as early as 6 hours after treatment with 80% methanol extract.

Event of apoptosis was confirmed quantitatively using flow cytometer machine. The cells were labelled with Annexin V-FITC and/or propidium iodide (PI) dyes. Six hours and 24 hours were selected to evaluate how soon the extract is able to induce apoptosis.  Figure 4 represents apoptosis event that occurs following treatment with 80% methanol of* A. crispa* extract.  Table 5 shows the percentage (%) of distribution of apoptosis events before and after treatment. The cells were differentiated into four quadrates based on the cell stages: viable, early apoptosis, late apoptosis, and necrosis quadrates.

At 6 hours and 24 hours of treatment, 80% methanol extract induced apoptosis to treated 4T1 mammary cell lines though without statistical significance (*P* > 0.05). The result shows that* A. crispa* are able to induce apoptosis as early as 6 hours following treatment with 80% methanol extract at their respective IC_50_ value. In control (untreated cells), there was no apoptosis event occurrence (0%) claiming that the plant is the source of apoptosis inducing event to occur. As the time of treatment increased to 24 hours, the percentage of apoptotic cells (early and late) also increased proportional to time of exposure.

## 4. Discussion

This study was aimed to investigate the potential cytotoxicity activity of* A. crispa* against* Mus musculus* mammary carcinoma cell line (4T1) in vitro. The leaves of* A. crispa* were extracted using 80% methanol, followed with solvent partitioning with ethyl acetate and aqueous extract. The cytotoxicity of the different extracts against 4T1 cells were assessed using cell based colourmetric assay, (3-(4,5-dimethylthiazol-2-yl)-2,5-diphenyltetrazolium bromide) MTT assay [20, 21]. The cytotoxicity classification was sorting based on United State National Cancer Institute (USNCI) plant screening program. IC_50_ value of extract that is between 20 and 100 *μ*g/mL is considered moderately cytotoxic [11, 22, 23] while less than 20 *μ*g/mL is considered strongly cytotoxic [23, 24].

Among 80% methanol, ethyl acetate, and aqueous extracts, 80% methanol extract showed the lowest IC_50_ value with 42.26 ± 1.82 *μ*g/mL. Analysis from the solvent partitioning revealed that ethyl acetate showed lower IC_50_ value with 52.41 ± 3.49 *μ*g/mL compared to aqueous extract which was 303.09 ± 48.09 *μ*g/mL. The results showed that as 80% methanol extract underwent solvent partitioning with ethyl acetate and aqueous extract, the IC_50_ values are getting higher indicating that cytotoxicity effect is weak. The 80% methanol extraction showed the best and most promising anticancer activity as compared to ethyl acetate and aqueous extracts. Further studies on mode of cell death of 80% methanol extraction were conducted.

In the present preliminary analysis for phytochemical compounds of 80% methanol extract from leaves, the results reported the presence of phenolic, flavonoid, saponin, tannin, terpenoid, and steroid. In the interest of narrowing down the phytochemical compounds with different polarity, the crude 80% methanol extract was further purified with solvent partitioning method with ethyl acetate (EA) and aqueous (AQ) extract. 80% methanol and ethyl acetate extracts revealed more phytochemical compounds as compared to aqueous extract. This result explained the weakest cytotoxicity effect exhibited by aqueous extract. The study conducted in [2] revealed that the root of* Ardisia *contains various phytochemical compounds such as phenolic, flavonoid, and saponin using hydroethanolic extract as solvent system. Results of this study also confirmed the findings of previous studies that pure alcoholic solvents like ethanol and methanol alone are not a preferable solvent system compared to the mixture of ethanol or methanol with water which is more effective especially in extracting phytochemical components from medicinal plants [25, 26].

Phytochemical compounds are plant secondary metabolites that are present in all plant species either in a mixture or alone but the amount differs between species. The phytochemical compound is not directly involved in plant growth and reproduction; however, their presence is crucially important in the plant defence systems against bacteria, fungus, virus, and free radicals. In application with that, the phytochemical compound of the plant has widely been extracted and used in healthcare products, cosmetics, and food flavouring industries [27]. Examples of the phytochemical compounds of plant origin which appeared to provide promising health benefit effects are phenolic, flavonoid, saponin, tannin, and terpenoid.

The phytochemical constituents that existed in* A. crispa *and its fractions play significant role in the ethnopharmacological medicinal values of the plants. Phenolics are the largest group of secondary plant metabolites that exhibits potent antioxidant and anticancer properties and is mostly found in plant-origin foods [28]. Flavonoids are a derivative of phenolic that have antitumour, antioxidant, and anti-inflammatory activity. Tannins have antiviral, antibacterial, and antiparasitic effects and anti-inflammatory, antiulcer, and antioxidant properties [29–31]. Saponins are known to have antimicrobial, anti-inflammatory [32], and anticancer activities [33].

80% methanol is known to be an efficient and widely used solvent system to extract natural antioxidative components, especially the phenolics, from plant materials. This is due to the fact that the methanol-water mixture has high polarity and thus greater efficacy towards the extraction of polar phytochemicals such as phenolics and flavonoids. Plants that contain high phenolic and flavonoid compounds are able to induce apoptosis and cell cycle arrest which is part of the mechanism of action [34]. The fact that 80% methanol extract of* A. crispa* has a good anticancer activity has been influenced by the selection of solvent. Similar results were also reported previously [25, 35, 36] and thus support this recent finding. The choice of extraction solvents such as water, acetone, ethyl acetate, alcohols (methanol, ethanol, and propanol), and their mixtures will influence the yields of phenolics extracted [37].

The results showed that as the 80% methanol extract undergoes partition with ethyl acetate and aqueous solution, the IC_50_ value is getting higher and selectivity index is getting lower which was not a good criterion of anticancer agent [38, 39]. 80% methanol extract was considered to have a low cytotoxicity to normal cells; meanwhile ethyl acetate extract was considered cytotoxic to both cancer and normal cells and aqueous extract was considered to have poor cytotoxicity effect. The 80% methanol extract of* A. crispa* can be classified as a good potential for antimammary cancer agent, safe, and generally not toxic to mammal cells when the selectivity index value is more than 2.0. The differences in CC_50_ among extracts could be due to certain phytochemical compounds that exist in the extract that act differently towards cells since phytochemical compounds that exist in plants are of multicomponent mixture. Thus, separation of the phytochemical compounds provides difference cytotoxic response [40]. It also can be influenced by the solvent used that makes some compounds soluble and some not soluble in particular solvent [41]. This is because phenolic compounds have broad secondary plant metabolites with many subgroups/classes and usually combined with other substances [40, 42]. It might be during partitioning of 80% methanol extract with ethyl acetate and aqueous extract that the chemical characteristic or solubility of phytochemical composition was altered. In a previous study, 6* Ardisia* species that have been evaluated (*A. crenata*,* A. escallonioides*,* A. mamillata*,* A. Japonica*,* A. compressa,* and* A. elliptica*) showed that the plant is significantly cytotoxic to hepatocellular carcinoma cell (HepG2) and the plant contains high phenolics compound [43]. In this current study, it is strongly believed that the mechanism underlying the anticancer effects was through the phenolics content. The plant that contains high phenolic and flavonoid compounds induces apoptosis and cell cycle arrest which is part of mechanism of action [34]. However, apoptosis assessment and cell cycle analysis are needed to be conducted in order to prove the mechanism of action.

Qualitative and quantitative methods were conducted to understand the mode of cell death and mechanism of action of the extract to cause cytotoxic effect to the mammary cancer cells. The antimammary cancer activity of extract was further supported with microscopic apoptosis evaluation by AO/PI dual fluorescent staining and quantification apoptosis analysis using Annexin V-FITC/PI staining. Furthermore, the morphological changes of 4T1 mammary cancer cells before and after treatment observed under inverted fluorescence microscope also support the finding grossly. The qualitative and quantitative analyses of apoptosis event are significantly important in order to determine and confirm the mechanism of action played by the extract [44].

Apoptosis is an important criterion for a potential anticancer agent. To confirm the detailed apoptosis event of the extract, flow cytometry analysis was performed. The cells were labelled with Annexin V-FITC and propidium iodide. Annexin V-FITC is a type of dye that has high affinity towards phosphatidylserine (PS) in the plasma membrane of cell. During apoptosis event, the plasma membrane of the cells becomes asymmetrical and loses its integrity, thus resulting in translocation of PS to the outer plasma membrane. This condition can be detected with Annexin V-FITC which is a type of calcium-dependent phospholipid-binding proteins and allowed permeability of dyes to PS and emits fluorescent colour which can be counted by flow cytometry machine. Since Annexin V is typically used for early apoptosis event, using it in conjunction with propidium iodide (PI) is really recommended for identification of early and late apoptotic cells because dead cells are permeable to PI. According to [45], most chemotherapeutic drugs are able to induce apoptosis within 3–24 hours. Interestingly, in this study, even though 80% methanol extract was considered as an extract, it was able to induce apoptosis event this fast, within 6–24 hours and in comparison to other chemotherapeutic drugs.

The regulation of apoptosis plays an important role in the development of antineoplastic drug. Imperfection of apoptosis modulation would impair body defence system and can initiate cancer development or continues cancer cell proliferation. No studies had been conducted on the apoptosis pathway specifically from the leaves of* A. crispa on *mammary cancer but previous study from [46] showed that ardisin (type of alkylphenols) from root extracts of* Ardisia brevicaulis *demonstrated cytotoxic effect against human lung cancer cells by inducing apoptosis pathway with G1 phase cell cycle arrest. Reference [43] stated that 6 species of* Ardisia* (*A. Compressa, A. Japonica, A. Elliptica, A. Crenata,* and* A. mamillata*) have cytotoxicity against human liver cancer (HepG2) through apoptosis mechanism. Hence, the results from previous studies support the current findings, linking phenolics and cytotoxic activity of* Ardisia crispa* with antimammary effect. Therefore, comprehensive studies are required intensively involving animal and clinical studies in order to elucidate the true potential of this plant.

## 5. Conclusion

Based on the result of this study, 80% methanol extraction of* A. crispa* can be considered as good antimammary cancer agent with high selectivity index. The high selectivity index showed ideal characteristic of anticancer agent, claiming that the 80% methanol extract of A*. crispa* is able to recognize between cancer and normal cells before killing it. These findings indicate the potential medicinal value of* A. crispa *in terms of cancer therapy and prevention. In vivo study and isolation of bioactive compound would provide more validation for impetus studies associated with inhibition and apoptotic effects against mammary cancer.

## Figures and Tables

**Table 1:** Percentage (%) of cell viability of leaves extract of *A. crispa* on 4T1 mammary cell lines after 72 hours of treatment.

Concentration (*µ*g/mL)	15.63	31.25	62.5	125	250	500	1000
80% methanol extract	88.00 ± 3.12^*∗*^	60.63 ± 1.97^*∗∗∗*^	31.16 ± 1.40^*∗∗∗*^	13.35 ± 1.51^*∗∗∗*^	11.58 ± 0.21^*∗∗∗*^	12.95 ± 4.34^*∗∗∗*^	11.14 ± 2.13^*∗∗∗*^
Ethyl acetate extract	97.08 ± 5.73	82.59 ± 8.76^*∗∗*^	34.81 ± 4.42^*∗∗∗*^	8.98 ± 0.43^*∗∗∗*^	9.33 ± 0.22^*∗∗∗*^	9.58 ± 1.40^*∗∗∗*^	9.07 ± 2.25^*∗∗∗*^
Aqueous extract	97.37 ± 1.17	91.59 ± 3.80	89.03 ± 6.44^*∗*^	66.13 ± 2.99^*∗∗∗*^	54.35 ± 5.34^*∗∗∗*^	38.26 ± 2.08^*∗∗∗*^	4.91 ± 2.64^*∗∗∗*^

Values are expressed as mean ± standard deviation (*n* = 3) per plate for three times' experiments. The comparison between treated cells and untreated cells (control) was evaluated using one-way ANOVA followed by Dunnett's multiple comparison test. ^*∗*^*P* < 0.05, ^*∗∗*^*P* < 0.01, and ^*∗∗∗*^*P* < 0.001 denote significant difference as compared to untreated cell (control).

**Table 2:** Percentage (%) of cell survivability of leaves extract of *A. crispa* on normal mouse fibroblast cell line (NIH3T3) after 72 hours of treatment.

Concentration (*µ*g/mL)	15.63	31.25	62.5	125	250	500	1000
80% methanol extract	100.28 ± 5.05	97.13 ± 6.42	92.46 ± 4.00	86.78 ± 9.43	67.34 ± 20.53	47.18 ± 2.52^*∗∗∗*^	36.81 ± 16.54^*∗∗∗*^
Ethyl acetate extract	97.08 ± 5.73	82.59 ± 8.76^*∗*^	34.81 ± 4.42^*∗∗∗*^	8.98 ± 0.43^*∗∗∗*^	9.33 ± 0.22^*∗∗∗*^	9.58 ± 1.40^*∗∗∗*^	9.07 ± 2.25^*∗∗∗*^
Aqueous extract	93.79 ± 9.56	76.48 ± 2.84	70.43 ± 2.33^*∗∗∗*^	63.36 ± 2.51^*∗∗∗*^	48.29 ± 2.42^*∗∗∗*^	35.93 ± 2.50^*∗∗∗*^	34.99 ± 0.77^*∗∗∗*^

Values are expressed as mean ± standard deviation (*n* = 3) per plate for three times' experiments. The comparison between treated cells and untreated cells (control) was evaluated using one-way ANOVA followed by Dunnett's multiple comparison test. ^*∗*^*P* < 0.05 and ^*∗∗∗*^*P* < 0.001 denote significant difference as compared to untreated cell (control).

**Table 3:** IC_50_ and CC_50_ and selective index of 80% methanol, ethyl acetate, and aqueous extracts.

Types of extract	IC_50_ on 4T1 (*µ*g/mL)	CC_50_ on NIH3T3 (*µ*g/mL)	SI
80% methanol	42.26 ± 1.82	431.94 ± 93.11	10.22
Ethyl acetate	52.41 ± 3.49	50.21 ± 1.35	0.96
Aqueous	303.09 ± 48.08	236.42 ± 16.94	0.78

Values are expressed as mean ± standard deviation (*n* = 3) per plate for three times' experiments.

**Table 4:** Phytochemical constituent of different extracts of *A. crispa* leaves.

Phytochemical constituent	Sample	Result
Phenolics	Hydromethanol extract	++
	Ethyl acetate extract	+++
	Aqueous extract	+

Flavonoids	Hydromethanol extract	++
	Ethyl acetate extract	+++
	Aqueous extract	+

Saponins	Hydromethanol extract	++
	Ethyl acetate extract	+
	Aqueous extract	−

Tannins	Hydromethanol extract	++
	Ethyl acetate extract	+++
	Aqueous extract	+

Terpenoids	Hydromethanol extract	+
	Ethyl acetate extract	++
	Aqueous extract	+

For phenolic, flavonoids, tannin, and terpenoids, −: no colour; +: weak colour; ++: moderate colour; +++: intense colour.

For saponin, −: no froth for 10 minutes; +: 1-2 cm froth maintained for 5 minutes; ++: 2-3 cm froth maintained for 10 minutes; +++: 3 cm and more froth maintained for 10 min.

**Table 5:** Percentage (%) of viable cells, early apoptosis, late apoptosis and necrosis of 4T1 cells following treatment at their respective IC_50_ after 6 and 24 hours.

	Percentage (%) of 4T1 mammary cells at different time points			
	Viable cells	Early apoptosis	Late apoptosis	Necrosis
Control (untreated)	98.00 ± 2.08	0.17 ± 0.15	0.00 ± 0.00	1.87 ± 2.11
IC_50_ at 6 hours	81.30 ± 2.26	8.30 ± 1.11	0.70 ± 0.42	9.70 ± 4.95
IC_50_ at 24 hours	30.90 ± 1.61	0.8 ± 0.10	3.82 ± 1.44	30.10 ± 0.26

All data are expressed as mean ± SD (standard deviation).
